# Different increase rate in body mass of two marten species due to climate warming potentially reinforces interspecific competition

**DOI:** 10.1038/s41598-021-03531-1

**Published:** 2021-12-17

**Authors:** Anna Wereszczuk, Tim R. Hofmeester, Alexander Csanády, Tomislav Dumić, Morten Elmeros, József Lanszki, Aksel B. Madsen, Gerard Müskens, Malamati A. Papakosta, Marcin Popiołek, Margarida Santos-Reis, Iñigo Zuberogoitia, Andrzej Zalewski

**Affiliations:** 1 Mammal Research Institute, Polish Academy of Sciences, Białowieża, Poland; 2 Resource Ecology Group, Wageningen University, Wageningen, The Netherlands; 3 Department of Wildlife, Fish, and Environmental Studies, Swedish University of Agricultural Sciences, Umeå, Sweden; 4 Department of Biology, Faculty of Humanities and Natural Sciences, University of Prešov, Prešov, Slovakia; 5 Department of Wildlife Management and Nature Conservation, Karlovac University of Applied Sciences, Karlovac, Croatia; 6 Department of Bioscience, Kalø, Aarhus University, Roende, Denmark; 7 Carnivore Ecology Research Group, Szent István University, Kaposvár, Hungary; 8 Animal Ecology Team, Environmental Sciences Group, Wageningen University & Research, Wageningen, The Netherlands; 9 Lab of Wildlife & Freshwater Fisheries, Department of Forestry and Management of the Environment and Natural Resources, Democritus University of Thrace, Orestiada, Greece; 10 Department of Parasitology, University of Wrocław, Wrocław, Poland; 11 Faculdade de Ciências, Centre for Ecology, Evolution and Environmental Changes (cE3c), Universidade de Lisboa, Campo Grande, 1749-016 Lisbon, Portugal; 12 Estudios Medioambientales Icarus S.L., Bilbao, Spain

**Keywords:** Ecology, Ecology, Environmental sciences

## Abstract

Many species show spatial variation in body size, often associated with climatic patterns. Studying species with contrasting geographical patterns related to climate might help elucidate the role of different drivers. We analysed changes in the body mass of two sympatric medium-sized carnivores—pine marten (*Martes martes*) and stone marten (*Martes foina*)—across Europe over 59 years. The body mass of pine marten increased with decreasing latitude, whereas stone marten body mass varied in a more complex pattern across its geographic range. Over time, the average body mass of pine martens increased by 255 g (24%), while stone marten by 86 g (6%). The greatest increase of body mass along both martens’ geographic range was observed in central and southern Europe, where both species occur in sympatry. The body mass increase slowed down over time, especially in allopatric regions. The average pine/stone marten body mass ratio increased from 0.87 in 1960 to 0.99 in 2019, potentially strengthening the competition between them. Thus, a differential response in body size to several drivers over time might have led to an adaptive advantage for pine martens. This highlights the importance of considering different responses among interacting species when studying animal adaptation to climate change.

## Introduction

An animal’s body size is an important trait determining many aspects of its life and population dynamics through the differentiation of reproductive success and survival across changing biotic and abiotic conditions^1^. Consequently, there are many factors that influence the body size of a species such as climate, food availability—e.g., as a result of variation in site productivity—and the presence of predators or competitors^2,3^. Due to the importance of body size for the physiological processes associated with thermoregulation, many species show geographical patterns in body size related to climate^4–8^. These patterns have led to the proposition of e.g. Bergmann’s rule: the body size of endothermic vertebrates increases from warmer to colder climates to facilitate heat conservation^4–9^. Although recent studies have questioned the generality of Bergmann’s rule^10^, several birds and mammals follow it^11,12^, or show the exact opposite trend, being smaller in colder regions^12,13^. For species that show patterns in body size related to geographical differences in climate, one would expect them to respond in a similar way to changes in temperature over time^14,15^. Indeed, several endothermic taxa showed changes in body size as a response to climate warming similar to what would be expected based on geographic patterns^16–19^. In line with Bergmann’s rule, a rising temperature over time should cause animals to shrink in body size^14,15^. This animal body size decline with climate warming has been suggested as the third response to global warming, alongside range shifting and changes in phenology^2,16–19^. Conversely, for species which show a significant tendency opposite to Bergmann’s rule (smaller body size in low-temperature areas), an increase in body size should be expected with climate warming. However, studies investigating species responses to both geographical and temporal patterns in climate are so far limited.

Climate change does not occur uniformly across the biomes and continents, and it can affect animals to various extents. In Europe, the rate of climate change is increasing to the north and to the east^20^. Species responses differ according to a climate change velocity gradient (e.g. as found for shifts in species distributions and adaptation to new climate regimes^21,22^). Consequently, it is likely that changes in body size are faster in areas with a more rapidly changing climate. At the same time, animal body size changes may be limited by other factors, e.g. the possibility to dissipate excess heat because an increase in temperature causes an increase in need for water and in the energy costs of thermoregulation^23,24^ or interspecific competition^25^. For this reason, changes in body size should be faster in the initial stages of climate warming but should slow down over time, even if climate warming accelerates, due to other limiting factors^26,27^.

Although large geographical studies allow for the correction for some factors that changed over time independent of climate, there might be secondary effects of factors changing with climate. In Europe, forest productivity has increased, likely as a response to rising temperatures^28^. Such a rise in productivity has likely resulted in an increased food availability for most terrestrial vertebrates, which could lead to increased body condition^29^, and hence body size. Thus, regardless of body size responses to climate warming due to thermoregulation mechanisms, animals might respond to secondary changes in terms of food availability as a response to climate change. However, increases in food availability might also lead to increased densities of species, and thus increased competition over resources^30^.

A species’ capacity to adapt body size as a response to changes in climate or food availability is restricted by the presence of competitors^31^. An increasing similarity in body size between two species strengthens the competition between them^32^. Thus, the presence of a larger competitor could limit a species’ potential to increase body size as a response to increased food availability. Conversely, species differences in body size response to climate change can affect the competitive interactions of species occupying a similar niche^31,33^. Thus, the presence or absence of similar sized competitors should be considered when studying changes in body size as a potential response to climate warming.

Pine martens (*Martes martes*) and stone martens (*Martes foina*), are two morphologically and ecologically similar medium-sized carnivores (average body mass: 1.2 kg^34^), which co-occur over large parts of Europe. While pine marten are referred to as a woodland-dwelling species which inhabit various types of forests^35^, stone marten often utilize anthropogenic areas with human settlements^36^. However, both species often occur in sympatry in agricultural landscapes with a mosaic of small forest and field patches, where they forage on the same prey but stone marten locates resting sites in urban areas or open habitat, whilst pine marten are almost exclusively found in forest^37,38^. The use of anthropogenic areas by stone marten potentially reduces thermal constraints which may lead to a less pronounced response to climate warming. These differences in behaviour and ecology of both species may result in various responses of body size to spatial and temporal climate change. At a large scale, pine martens show an opposite trend to that predicted by Bergmann’s rule^12,13^: they are smaller in the colder climates of north-eastern Europe and larger in the warmer climates of south-western Europe^13,39^. In contrast to pine martens, stone martens show more complex patterns and a less pronounced geographical variation in terms of increase in body size from western to eastern Europe^13^ or from south to north^40^. Consequently, we hypothesize (1) that pine marten increased in body mass from the north to the south of Europe, opposite to Bergmann’s rule, while stone marten did not. Because of the increased productivity in European forests due to climate warming^28^, we expect both pine and stone marten to have increased in body mass, with a greater extent of increase toward the north according to climate change velocity. We thus hypothesize (2) pine marten to have had a larger increase in body mass over time (as a combined response to a temperature and productivity rise) compared to the stone marten (only responding to rise in productivity), which should also show an increase but less pronounced. Due to these different changes over time, we expect the difference in body mass between the species to have become smaller, potentially increasing interspecific competition. Furthermore, we hypothesize (3) that the presence of stone martens limited the adaptive capacity of pine martens, resulting in a lower body mass increase in sympatric areas compared to allopatric areas.

## Results

### Geographical and temporal body size variability

The mean body mass of pine martens was 1031 g (range 639–1500 g) for females and 1451 g (range 788–2200 g) for males, while the mean body mass of female stone martens was 1279 g (range 602–1930 g) and 1620 g for males (range 800–2500 g). We found support for an influence of all five explanatory variables (sex, month, year, latitude, and longitude) on the body mass of both marten species. The GAMs explained 67.4% and 42.2% of the variance in the response variables for pine and stone martens, respectively. Body mass differed between sexes in both species—males were significantly larger than females (Table 1, Supplementary Fig. A5). Body mass also varied over the months in both species (Table 1, Supplementary Fig. A5)—average body mass increased significantly from February to April, decreased in May to July, and again increased in September to October (Supplementary Fig. A5).

**Table 1:** Results of GAM analyses of the sex and smoothed effects of month and three-way interaction with latitude (Lat), longitude (Lon) and time (year) testing for the spatial and temporal gradient and related body mass of pine and stone martens in Europe.

Variables	Pine martens (N = 1658)				Stone martens (N = 2150)			
	Estimate	SE	t	p	Estimate	SE	t	p
**Parametric terms**								
Intercept	1031.21	7.26	142.03	< 0.001	1280.13	7.49	170.90	< 0.001
Sex (male)	419.49	8.99	46.67	< 0.001	338.59	10.13	33.43	< 0.001

	EDF max	EDF	F	p	EDF max	EDF	F	p
**Smooth terms**								
s (Lat, Lon, year)	32.09	40.34	13.35	< 0.001	38.01	46.89	4.26	< 0.001
s (month)	6.09	8.0	9.64	< 0.001	5.52	8.0	8.40	< 0.001

**Figure 1:**
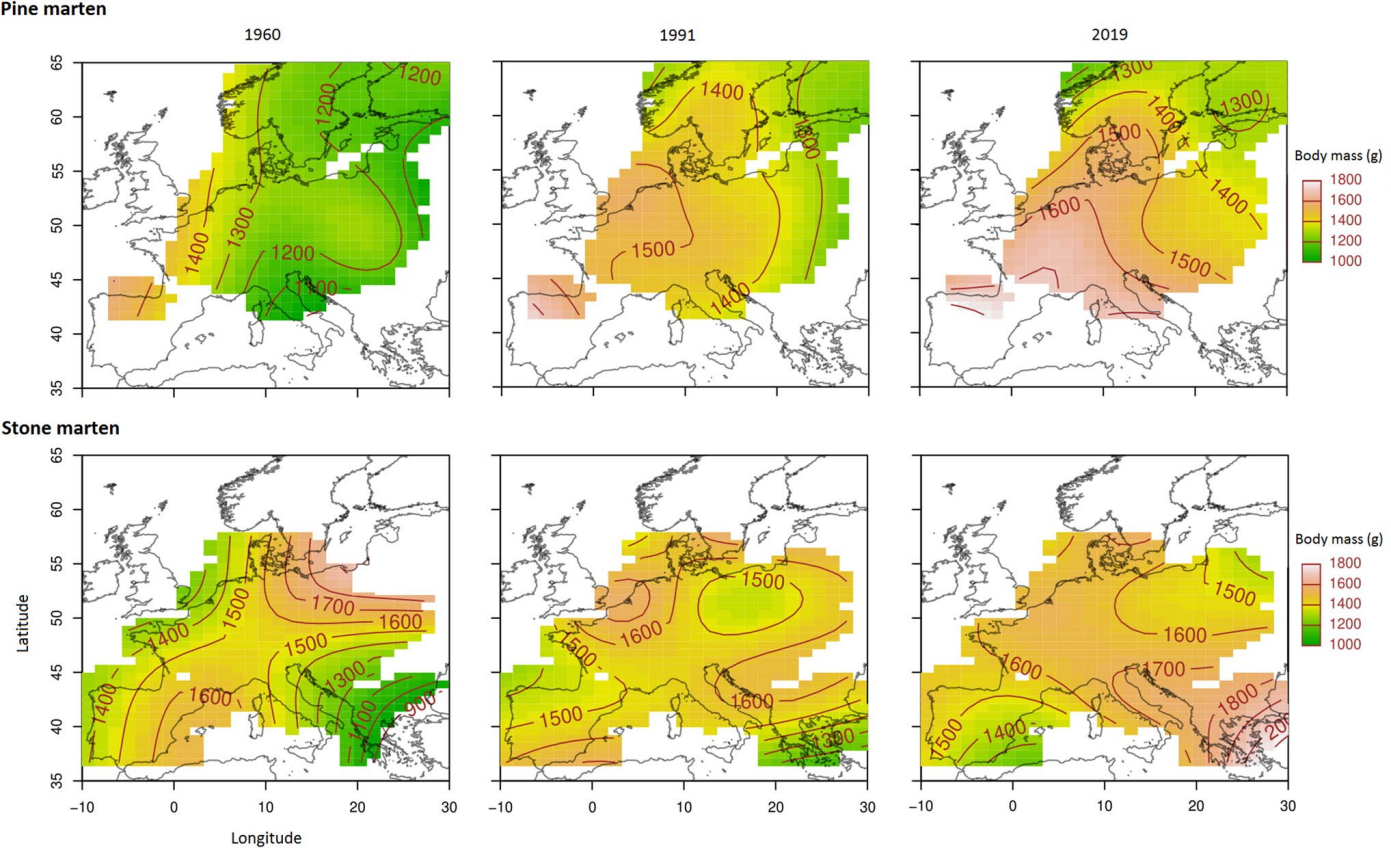
Spatial gradients in body mass of pine and stone marten males in Europe based on results of GAMs. Maps were produced for 3 years with approximately 30-year intervals (1960, 1991, and 2019). Interpolated lighter oranges and reds indicate greater body mass in grams; darker greens indicate lower body mass.

**Figure 2:**
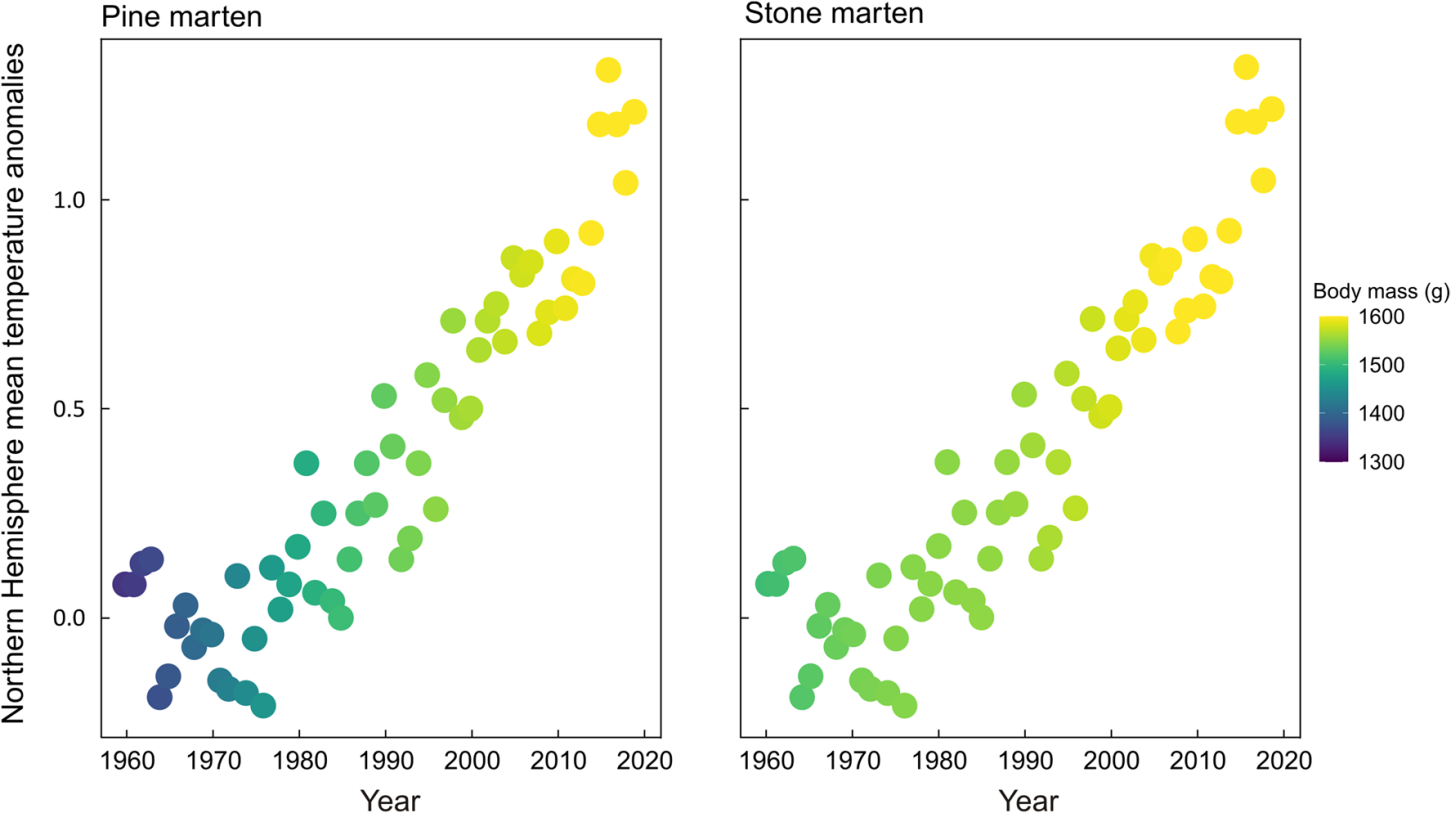
Northern Hemisphere mean temperature anomalies since 1960 and annual mean body mass of pine and stone marten males in Europe. Body mass changes are indicated by colour gradients.

Over the past 59 years (1960–2019), the body mass of both marten species increased (GAM three-way interaction between the time, latitude and longitude; Fig. 1) and this period was positively correlated with global temperature increases (expressed as Northern Hemisphere mean temperature anomalies; r = 0.916, p < 0.01; Fig. 2). During the last 59 years, the average body mass of pine martens increased by 254.7 ± 19.6 g (24%) and varied from 916.4 to 1344.5 g in 1960 and from 1100.3 to 1595.7 g in 2019, while stone marten body mass increased by 85.9 ± 38.6 g (6%) and varied from 1210.3 to 1594.3 g in 1960 and from 1217.9 to 1647.3 g in 2019. The average annual increase of body mass was 4.3 g (± 0.2 SE) for pine and 2.7 g (± 0.2 SE) for stone martens. The average difference between the lowest and the highest body mass across the geographical range (averaged among years) was 426.2 g (± 3.6 SE) for pine and 260.7 g (± 5.0 SE) for stone martens.

**Figure 3:**
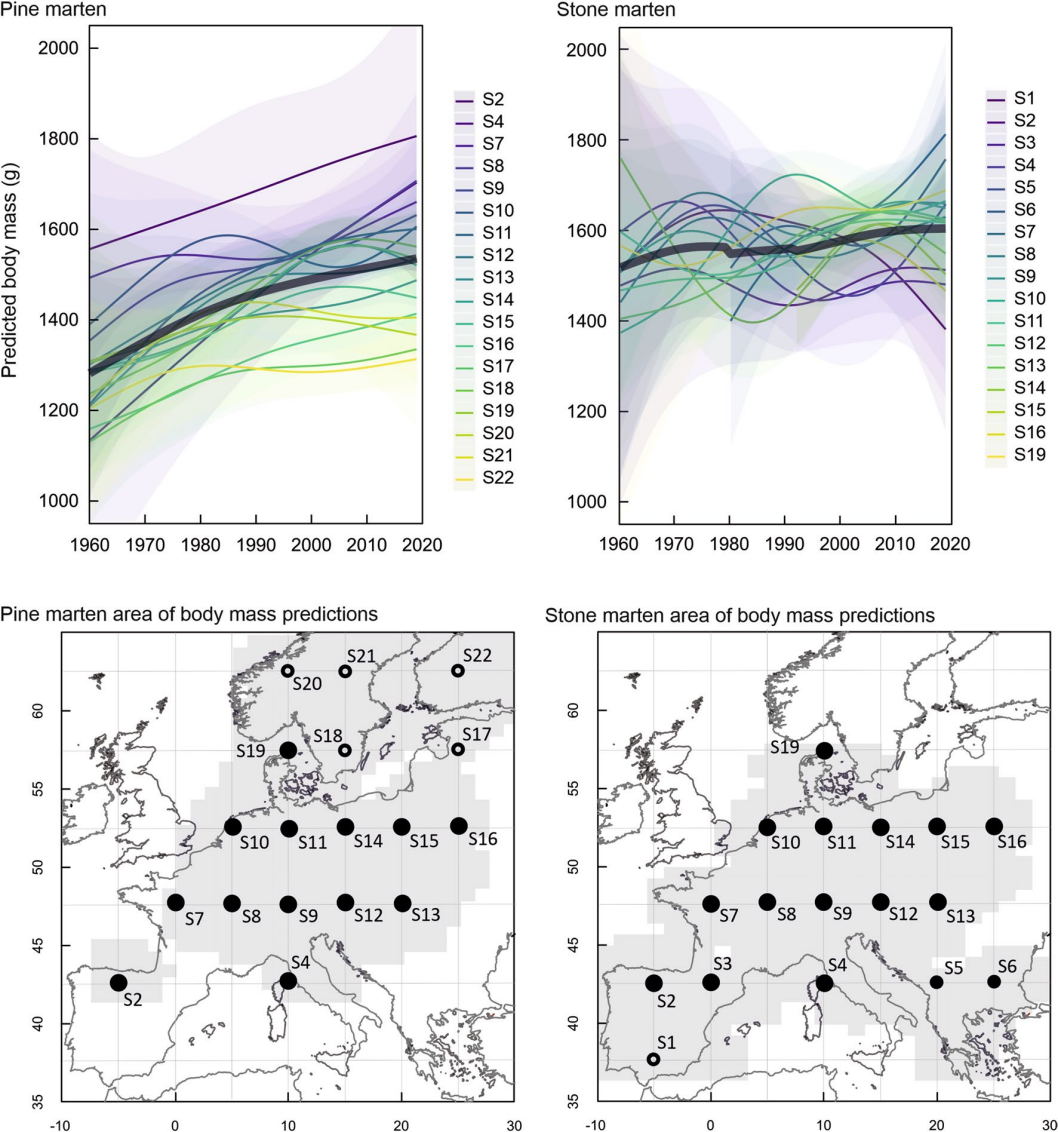
Body mass variation (with 95% confidence interval) of pine martens (*Martes martes*) and stone martens (*Martes foina*) in geographical gradient over time. Predictions are shown for males, predicted values for females are in Supplementary Table A2. Grey background on maps represents the geographic extent of GAM predictions of body masses based on collected data. Predicted body mass was visualized for sites of the geographical net with resolution 5° × 5° of latitude and longitude. Full dots represent sympatric sites while empty dots mean allopatric sites.

The body mass of both marten species also greatly varied in relation to geographical location (Fig. 3). Pine marten body mass increased from north-east to south-west and increased over time along the whole geographic range in all geographical locations. In contrast, stone marten body mass varied in a more complex pattern for geographical range and time. In south-eastern Europe, the body mass of stone martens increased over time, whereas in south-western Europe, the body mass varied non-linearly over time and even decreased in the southern Iberian Peninsula (Fig. 1). Generally, pine marten body mass increased in each site over the last 59 years, while stone marten body mass increased in 13 and decreases in 4 sites (S1, S3, S14, and S16) (Fig. 3). Pine marten body mass increased by 5–17% (58.5–178.9 g) in the northernmost populations, (S20–S22, Fig. 3), 15–42% (166.3–420.5 g) in central part of its range, (S7–S19), and 18–62% (251.2–572.9 g) in the southern edge of its range, (S2 and S4). Stone marten body mass increased by 10–33% (132.5–410.9 g) in most of its range (S4–S10, S12, S13 and S19, Fig. 3, Supplementary Table A2), with the exception of S11 where body mass increased only slightly, by 1% (19.9 g). Slight increase by up to 3% (34.3 g) or decrease of up to 13% (209.6 g) was observed in areas located at the extremes of its distribution (south-west: S1–S3 and north-east: S14–S16).

### The body mass ratio of the two marten species

**Figure 4:**
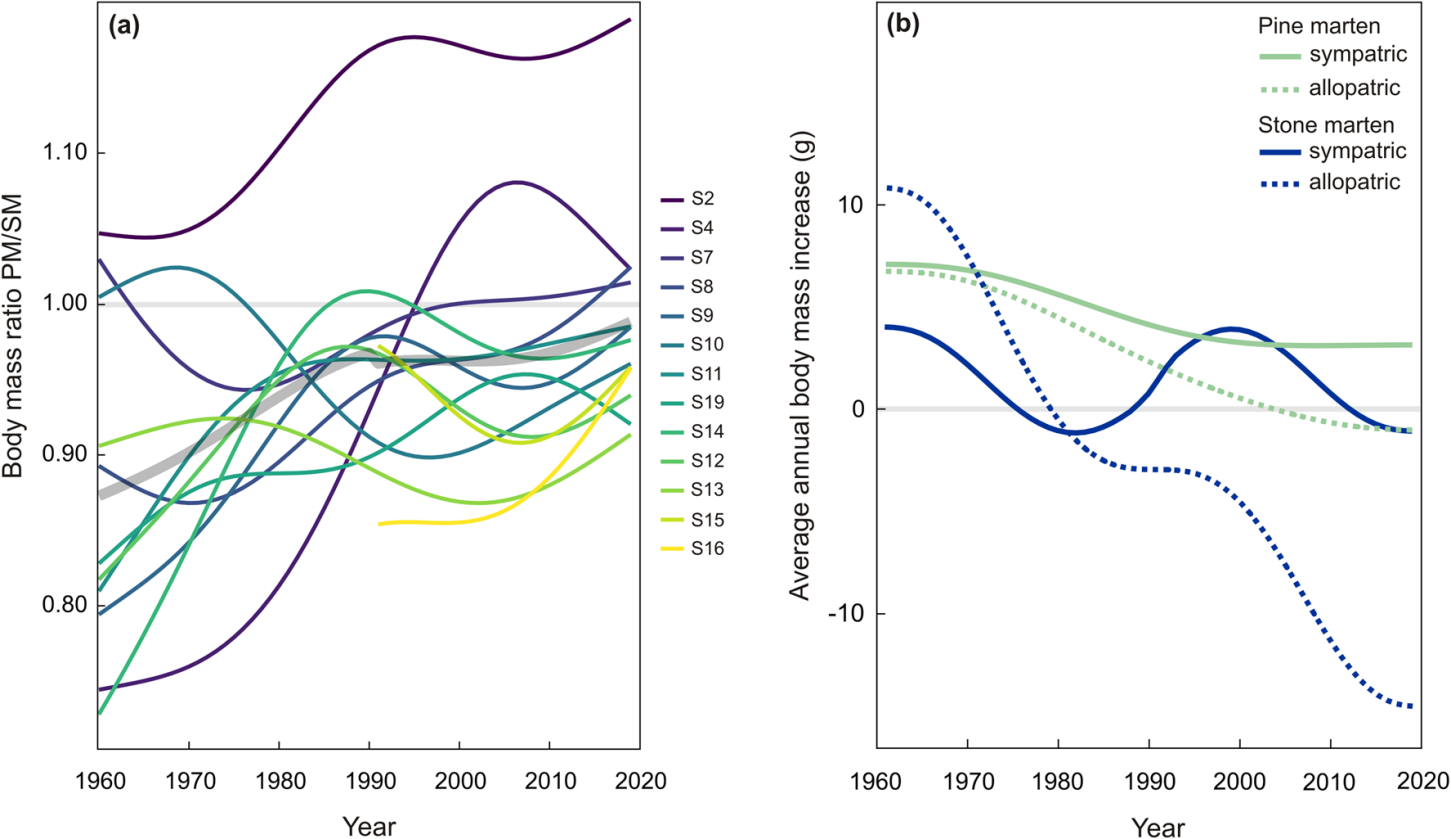
Changes of the ratio between pine marten (PM) and stone marten (SM) body mass in Europe over 59 years (**a**); bold grey line represents body mass ratio PM/SM averaged for sites. Mean annual predicted body mass increase averaged for sympatric and allopatric sites (**b**). Stone marten allopatric area was represented by one site in southern Spain (S1); site’s location in Fig. 3.

An average body mass ratio between pine and stone martens (PM/SM) was calculated for 13 sites where both species occurred in sympatry. Except for one site, the mean ratio was below 1—stone martens were heavier than pine martens, and this varied in geographical scale between 0.88 in S16 (north-eastern edge of its range) and 1.13 in S2 (the northern Iberian Peninsula) (averaged among years; Supplementary Table A3). The average PM/SM ratio increased in consecutive years from 0.87 in 1960 to 0.99 in 2019 (Fig. 4a) indicating that the body mass of both species became more similar over time. Over the complete study period of 59 years, the PM/SM ratio exceeded 1 at some point in time in five sites (Fig. 4a).

The mean annual increase in body mass differed in the sympatric and allopatric areas for both species (Fig. 4b). Pine marten body mass annually increased by 4.77 ± 0.14 g in sympatric areas, while in allopatric areas, it increased by 2.74 ± 0.26 g, and over the full study period, the body mass increase of pine martens was higher in sympatric areas (Fig. 4b). Stone marten body mass increased annually by an average of 1.29 ± 0.20 g in sympatric areas and decreased by 2.31 ± 0.70 g in the allopatric part of the range (site S1; Fig. 4b). However, care has to be taken in the interpretation of the results in allopatric areas as the number of sites was small and all sites were located at the extremes of the distributions of both species.

## Discussion

Using data from a large database of body mass measurements distributed over a vast geographical area and a period of almost 60 years, we found that pine marten body mass decreased with increasing latitude and increased with time. A similar trend for both spatial and temporal variation in pine marten body size suggests that the same factor or combination of factors drive these changes. In contrast, geographic variation of stone marten body mass showed a more complex spatio-temporal pattern that varied across regions. These different rates of body mass changes occurring over time and space in both marten species affected the body mass ratio between the species and may have increased interspecific competition in local populations. A higher body mass increase in sympatric areas compared to allopatric areas contradicted our prediction that body mass increase was limited by interspecific competition. This might have been caused by confounding patterns in climate change intensity and productivity increase, with continued limited productivity in the allopatric part of the stone marten range. In addition, pine marten body mass increase was lower in the allopatric area, despite a greater productivity increase and temperature warming than in the sympatric area, which is also contrary to our predictions. Therefore, our results suggest the importance of interspecific competition in body mass changes in response to climate change.

Body mass is commonly used as an index of animal body size^41^, but this metric also includes body condition, which varies within an individual’s lifetime and may give different results than a structural size metric only^42^. However, martens have limited possibility to accumulate fat reserves and they rely little on body energy reserves^43^. Thus, we expect that a significant portion of body mass increase will be due to an increase in structural body size. This prediction was confirmed by a recent study suggesting increase in a skull morphometry, as a proxy of body size, in pine marten^44^. The study spanned the last 117 years, but an increase in body size had occurred from around 1960, exactly in line with the results of this study. Therefore, we assume that the body mass increase at a large geographical scale obtained in this analysis resulted largely from a structural size increase and only in minor part from an increase in body condition.

The increase in body size over time may result from two mechanisms: (1) an increase in productivity can provide an increase and more stable food base that causes an increased growth rate in juvenile animals and the increased survival of larger individuals and/or (2) a release from the constraint of extreme winter temperature increases the survival of larger individuals. In several species, the adult body size is a result of the length of the period when high-quality food is taken during growth^45,46^. Thus the mean increase in body mass of both pine and stone marten over time might have been caused by the observed increase of primary production and food abundance appearing with climate warming at mid- and high-latitudes^47^. The increasing abundance of berry shrubs in forests as a result of climate change^48^ may enhance the availability of main pine marten prey^49^ due to the positive correlation between berry and rodent densities^50,51^. Higher rodent density may positively affect pine and stone marten body mass. This suggestion was confirmed for the American marten (*Martes americana*), which increased in size between 1949 and 1998 in Alaska. The increase was explained by lower energy expenditure and improved food availability, considered as the increase in annual net primary production, which led to larger rodent populations^52^. Besides climate change, food availability can be affected by land use changes over time, such as deforestation and habitat fragmentation^53^.

Secondly, larger individuals may be released from low temperature constrains due to climate change. Winter thermal stress is the most important factor affecting the duration of pine marten daily activity^54^. To optimize their energy budget and reduce heat loss at low temperatures, pine martens reduce activity and select well-insulated resting sites during the day, while satisfying their food requirements by shifting to feed on ungulate carrion and/or hunting larger prey species and storing the food^54,55^. Then, a smaller body probably results in higher survival due to lower energy needs^56,57^. Increasing winter temperatures may release pine martens from this restriction, and larger individuals may potentially survive winters more often, which could have caused the increase in body size over time. Built-up areas utilised by stone marten provide well-insulated resting sites and additional food sources (e.g. rodents, food waste, fruits) within buildings, which allow stone martens to avoid the pressure of severe weather conditions. A recent study found that body mass of mammals was greater in areas with higher index of urbanization^58^. Stone marten body size could have already been higher during past periods of colder climate and increase only slightly with climate warming.

Summarizing, both potential reasons for the increase in martens body mass over time, an increasing productivity and a release from winter temperature constraints, are not mutually exclusive. Higher food abundance allows an increased rate of growth in juvenile individuals which can then achieve a larger body size. At the same time, warming temperatures allow martens with larger body sizes to survive the winter more easily, when larger individuals would otherwise need to maintain a greater body size while also reducing activity to avoid exposure to the lowest temperatures.

The increase in body size across the geographical scale (over space) seems to be determined by the same force affecting temporal variation in body size of the studied species. In our dataset, the body mass of pine martens increased towards the south, consistent with previous analyses of geographical variability of this species showing that pine martens are larger in warmer geographical regions^13,39^. We found a less clear spatio-temporal pattern of body mass variability for stone marten, which was more ambiguous and the increase of body mass over time varied between locations. These spatial variations might be due to (1) the stone marten inhabiting various habitats in different locations, (2) the ongoing range and demographic expansions of stone martens in some locations in Europe, and (3) the varying competitor community structure across the stone marten range. In western Europe, stone martens inhabit natural habitats^36^, and their body mass highly increased over time in this region. In central Europe, stone martens inhabit cities, villages, and fragmented forest-field landscapes^36^. In these habitats, stone marten body size could vary due to, e.g., differences in food availability as well as in predation and competition pressure. Built-up areas provide well-insulated resting sites and additional food sources (e.g. rodents) within buildings, which allow stone martens to reduce their activity outside of buildings, and thus, avoid the pressure of severe weather conditions. It is possible that a body size threshold exists and it could have already been higher during past periods of colder climate and increased only slightly with climate warming. Furthermore, changes in agricultural production and management of the landscape over time might have improved the diet of carnivores commensal with humans and caused the increase in their body size^59^.

The body mass of stone martens decreased in two regions of its range. In north-eastern Europe, stone martens recently expanded their range^60^. At the beginning of a range expansion, larger animals can disperse, further colonizing new areas where, due to low density, intraspecific competition is low and body mass is larger^61,62^. The increase in stone marten density in this region might have increased intraspecific competition that could have caused a body mass decrease over time. Such a decrease of body mass, during a period of population establishment and demographic expansion, was observed in American mink (*Neovison vison*) and red fox (*Vulpes vulpes*) in Europe^63,64^. Stone marten body size also decreased on the southern Iberian Peninsula, but the mechanism driving this change is unclear. All these factors confounded our results to a varying extent, causing a weaker response of stone marten body size to latitudinal climate variation and temporal climate warming.

Our results show that the stone marten was heavier than the pine marten, except on the northern Iberian Peninsula, where the stone marten is lighter than the pine marten (see Fig. 4a, site S2). The co-occurrence of mesocarnivores is driven largely by the relative body size of the species, while trophic relationships or food availability seem to be less important^31^. Previous studies suggested that the stone marten can be outcompeted by the pine marten, especially in forested habitats and in Mediterranean climate^65–67^. The competitive advantage of the pine marten on the Iberian Peninsula is likely driven by a combination of bigger size and the occurrence of the common genet, which competitively displaced the stone marten from areas with dense vegetation^68^. However, in other parts of Europe, the stone marten generally had a competitive advantage in body mass over the pine marten. We found that the body mass of pine martens increased faster than that of stone martens over the last 59 years, and for pine marten this increase is greater in sympatric than allopatric areas despite the fact that climate is warming faster in allopatric areas (north Europe) than sympatric areas (central Europe)^69^. However, due to the limited allopatric range of both species, these results should be treated with caution. As a consequence of faster body size increase in sympatric areas, the body mass difference between both marten species has narrowed over time and even turned in favour of the pine marten in five sites. The release from a temperature constraint could probably promote the competitive advantage of larger pine martens, causing a further increase in pine marten size in areas where they occur alongside larger competitors. Such competitive advantage has previously been suggested, as polecat body size increased after the introduction of a larger competitor, the American mink^62^. The apparent similarity in stone and pine marten body mass may strengthen competitive interactions between both species.

## Conclusions

Our results showed a link between spatial and temporal body size changes in relation to climate change, related changes in productivity, and interspecific interactions. The pine marten, a species that showed a tendency opposite to Bergmann’s rule, increased in body mass as temperature increased over time. In a species for which the geographical variability of body mass was related to more complex processes, the stone marten, the changes in body mass over time were more ambiguous. Due to the confounding changes in climate, land-use and productivity over the past decades in Europe, it is hard to disentangle the separate effects of these drivers on the patterns we found. Because of the importance of body mass in driving species interactions with their environment, there is an important need to better understand the mechanisms driving body mass variation over space and time. Furthermore, we found several indications that interspecific competition influenced species’ capabilities to adapt to climate change. This highlights the importance of considering interactions among species when studying animal adaptation to climate change.

## Materials and methods

The data on the body mass of pine and stone martens were obtained from natural history museums, published papers, and data collected by the authors (listed in the Supplementary Table A1). These data were obtained from dead animals collected as road-killed and hunted animals or from live trapped individuals. Based on body mass distribution for each separate species and sex, we excluded 50 measurements of pine martens and 66 of stone martens that departed from the range of body mass for each group of marten (in total, 2.9% of the individuals were removed). Those measurements may have been taken from incorrectly identified species, individuals weighed without the skin, or from young martens weighed from March to June. Data collected from 1960 to 2019 were included in the analysis. Finally, we analyzed the body mass of 1658 pine and 2150 stone martens in Europe (Supplementary Figs. A1–A4).

We aimed to test long-term trends in body size variation across broad spatial gradients. For this purpose, we used generalized additive models (GAMs), which is the best approach for modeling long-term nonlinear trends^70^, using the ‘mgcv’ package version 8.1^71^ implemented in R version 4.0.0^72^. The GAMs were conducted separately for each species with latitude, longitude, year, month in which the animals were found dead or trapped, and sex as explanatory variables. The body mass includes body condition, which varies within an individual’s lifetime (lower in winter and higher in spring–summer) and may give different results than a structural size metric only^42^. To partially account for body condition variation across the seasons, we included the month in which the animals were found dead or trapped in the analyses. The latitude, longitude, and year were fitted as a three-way interaction using a tensor product of a thin-plate regression spline^71^. The smoothing using a tensor product addresses the issue of modeling responses to interactions of multiple inputs with different units. The upper limit of the degrees of freedom associated with each of the explanatory variables (latitude, longitude, and year) was set to 5 in all cases in each three-way interaction. Models were specified using Gaussian distribution, an identity link function, and a REML approach. Cyclic cubic regression splines were used for smoothing of months, as recommended for cyclic variables^71^. Spatial predictions from the models were restricted to avoid undue extrapolation, and mapped predictions were controlled within the range of the original covariate values using too.far = 0.10 (‘mgcv’ package). Comparison of body mass measurements of martens weighed alive (trapped individuals) and dead showed no difference between measurements types (GAM: p = 0.591 for pine and p = 0.309 for stone marten), so this variable was not included in further analyses.

To analyze different patterns in body mass variation over time across geographic locations, we calculated predicted body masses from the model for each species in regularly distributed sites. Based on GAM predictions, we selected eighteen sites in the geographical range of pine martens and seventeen sites in the range of stone martens in a geographical net with a resolution of 5° × 5° of latitude and longitude to provide a reliable representation of European biogeographical regions in the ranges of both species. Extreme points at which inferences may be less precise and those far from land were omitted. Because we did not have measured body mass for stone martens from all sites throughout the study period, we used shorter periods for some predictions: 1980–2019 for S5 and S6 and 1991–2019 for S15 and S16. After this, we estimated the predicted body mass ratio of pine marten to stone marten (PM/SM) for each sympatric and allopatric site.

We used Northern Hemisphere mean temperature anomalies as a proxy for global temperature changes. The data on anomalies were obtained from the National Aeronautics and Space Administration (NASA), Goddard Institute for Space Studies (https://data.giss.nasa.gov/gistemp; source: GHCN-v4 1880-04/2020; GISTEMP team, 2021)^73^, and were calculated relative to the mean temperature of the reference period 1951–1980. We correlated these annual anomalies with the predicted body mass averaged over the years.

## Supplementary Information

Supplementary Information.

## Data Availability

All collected data are provided in the Supplementary Information or available upon request directed to corresponding author.
