# Supplementary material for: Different increase rate in body mass of two marten species due to climate warming potentially reinforces interspecific competition
Source: Sci Rep. 2021 Dec 17;11:24164. doi: 10.1038/s41598-021-03531-1 (PMC8683469; doi:10.1038/s41598-021-03531-1)
Supplement: Supplementary file 1 — Supplementary Information. [file 41598_2021_3531_MOESM1_ESM.pdf]

APPENDICES

Different increase rate in body mass of two marten species due to climate warming potentially reinforces interspecific competition

Anna Wereszczuk[1*], Tim R. Hofmeester[2,3], Alexander Csanády[4], Tomislav Dumić[5], Morten Elmeros[6], József Lanszki[7], Aksel B. Madsen[6], Gerard Müskens[8], Malamati A. Papakosta[9], Marcin Popiołek[10], Margarida Santos-Reis[11], Iñigo Zuberogoitia[12], Andrzej Zalewski[1]

[1] Mammal Research Institute, Polish Academy of Sciences, Białowieża, Poland

[2] Resource Ecology Group, Wageningen University, Wageningen, Netherlands

[3] Department of Wildlife, Fish, and Environmental Studies, Swedish University of Agricultural Sciences, Umeå, Sweden

[4] University of Prešov, Department of Biology, Faculty of Humanities and Natural Sciences, Prešov, Slovakia

[5] Department of Wildlife Management and Nature Conservation, Karlovac University of Applied Sciences, Karlovac, Croatia

[6] Department of Bioscience, Kalø, Aarhus University, Roende, Denmark

[7] Carnivore Ecology Research Group, Szent István University, Kaposvár, Hungary

[8] Animal Ecology team, Environmental Sciences Group, Wageningen University & Research, Wageningen, Netherlands

[9] Department of Forestry and Management of the Environment and Natural Resources, Lab of Wildlife & Freshwater Fisheries, Democritus University of Thrace, Greece

[10] Department of Parasitology, Institute of Genetics and Microbiology, Wrocław University, Poland

[11] Centre for Ecology, Evolution and Environmental Changes (cE3c), Faculdade de Ciências, Universidade de Lisboa, Campo Grande 1749-016, Lisboa, Portugal

[12] Estudios Medioambientales Icarus S.L., Bilbao, Spain

Table A1. Museums, institutions and persons who shared data of pine marten *Martes martes* (Mm) and stone marten *Martes foina* (Mf) measurements.

Nr	Person	Institution	Number of individuals
1	Hermann Ansorge	part of data origin from the Senckenberg Museum of Natural History in Görlitz, Germany	Mf-13, Mm-18
2	Giorgio Giuseppe Bardelli	Natural History Museum in Milan, Italy	Mf-1
3	Ludovic Besson	Muséum Gabriel Foucher, Muséum d'histoire naturelle de la ville de Bourges, France	Mf-2, Mm-4
4	Celia Bueno	Muséum d'histoire naturelle de Neuchâtel, Switzerland	Mf-11, Mm-1
6	Sim Broekhuizen, Gerard Müskens	Animal Ecology team, Environmental Sciences Group, Wageningen University & Research, Wageningen, Netherlands	Mf-410, Mm-311
7	Tomislav Dumić	Department of Wildlife Management and Nature Conservation, Karlovac University of Applied Sciences, Karlovac, Croatia	Mf-42
8	Morten Elmeros	Department of Bioscience, Kalø, Aarhus University, Grenå, Denmark	Mf-318, Mm-87
9	Pablo Ferreras, Francisco Díaz-Ruiz	IREC, Spanish Game Research Institute, Spanish National Research Council; University of Malaga, Department of Animal Biology, Spain	Mf-17, Mm-5
10	Magnus Gelang	Gothenburg Natural History Museum, Sweden	Mm-24
11	Olivier Glaizot	Vaud Zoology Museum/Musée cantonal de zoologie, Lausanne, Switzerland. Specimen numbers: 20079, 30304, 42168, 42291.	Mf-4
12	Martti Hildén	Finnish Museum of Natural History, Helsinki, Finland	Mm-30
13	Ladislav Hlôška	Povazie Museum in Žilina, Slovakia	Mf-8
14	Tim R. Hofmeester	Resource Ecology Group, Wageningen University, Wageningen, Netherlands; Department of Wildlife, Fish, and Environmental Studies, Swedish University of Agricultural Sciences, Umeå, Sweden	Mf-35, Mm-50
15	Daniela Kalthoff	Department of Zoology, Swedish Museum of Natural History (NRM), Sweden	Mm-60
16	Ari Karhilahti	Zoological Museum of the University of Turku, Finland	Mm-10
17	Peter Krišovský, Aristid Mošanský	East Slovakian Museum in Kosice, Slovakia	Mf-16, Mm-12
18	Boris Kryštufek	Department of Slovenian Museum of Natural History, Slovenia	Mf-9, Mm-8
19	József Lanszki, Tamás Görföl	Kaposvár University, and part Hungarian Natural History Museum, Mammal Collection, Hungary	Mf-106, Mm-15
20	Stephan Liersch	Natural History Museum of the Grisons, Chur, Switzerland	Mf-17, Mm-5

21	Aksel Bo Madsen	Department of Bioscience - Kalø, Aarhus University, Aarhus, Denmark	Mf-153
22	Nicolas Margraf	Musée d'histoire naturelle de La Chaux-de-Fonds, Switzerland	Mf-1
23	Christian Montermann	Zoological Research Museum Alexander Koenig, Germany	Mf-24, Mm-2
24	Robert W. Mysłajek	Faculty of Biology, Institute of Genetics and Biotechnology, University of Warsaw, Poland	Mf-4
25	Malamati A. Papakosta	Department of Forestry and Management of the Environment and Natural Resources, Lab of Wildlife & Freshwater Fisheries, Democritus University of Thrace, Greece	Mf-119
26	Marcin Popiołek	Department of Parasitology, Institute of Genetics and Microbiology, Wrocław University, Poland	Mf-50, Mm-19
27	Miguel Proa	Museum of Natural Sciences of Angers, France	Mf-7
28	Manuel Ruedi	Museum d'histoire naturelle de Genève (Natural History Museum Geneva), Switzerland	Mf-37, Mm-4
29	Margarida Santos-Reis	Centre for Ecology, Evolution and Environmental Changes (cE3c), Faculdade de Ciências, Universidade de Lisboa, Campo Grande 1749-016, Lisboa, Portugal	Mf-51
30	Ursula Stockinger	Universalmuseum Joanneum, Abteilung Naturkunde, Zoologie, Austria	Mf-3
31	Voitto Takkunen	Private collection, Finland	Mm-436
32	Dieter Thomas Tietze	Naturhistorisches Museum Basel, Switzerland	Mf-15, Mm-1
33	Carlos Urdiales Alonso	Doñana Biological Station Vertebrate Collections, Spain. Specimen numbers: 1704, 1706, 1707, 1708, 1709, 1710, 1721, 1722, 2652, 3494, 3495, 3496, 3497, 6777, 16769, 20654, 21224, 21225, 21680, 24774, 24775, 25227, 25918, 25933, 25936, 26039, 26040, 26041, 26042, 26045, 26048, 26197, 26323, 27762, 27987, 28136, 28181, 28182, 28464, 28465, 28466, 30190, 30293, 30295, 30296, 30315, 30460, 30471, 30473, 30474, 30475, 2.012.085.021, 2.012.085.022, 2.012.085.023, 2.012.085.024, 2.012.085.025, 2.012.085.026, 18061, 19389.	Mf-59, Mm-7
34	Geraldine Veron	Muséum National d'Histoire Naturelle, Mammal Collection, Paris, France	Mf-4
35	Tibor Weisz, Alexander Csanády	Saris Museum Bardejov, Slovakia;<br>University of Prešov, Department of Biology, Faculty of Humanities and Natural Sciences, Prešov, Slovakia	Mf-9, Mm-86
36	Øystein Wiig	Natural History Museum, University of Oslo, Mammal Collection, Norway	Mm-45
37	Nuri Yiğit, Ercüment Çolak	AUMAC: Ankara University, Mammalian Research Collection, Turkey	Mf-12

38	Frank Zachos	Natural History Museum Vienna, Mammal Collection, Austria	Mf-42, Mm-5
40	Andrzej Zalewski, Anna Wereszczuk	Ecophysiology and Behavioral Ecology Research Unit, Mammal Research Institute PAS and Zoological Collection of the Mammal Research Institute PAS, Poland	Mf-150, Mm-137
41	Iñigo Zuberogoitia	Estudios Medioambientales Icarus S.L., Bilbao, Spain	Mf-22

Data obtained from the literature:

Bardonnet, C. (2015). Une analyse de la connectivité fonctionnelle du paysage: Etude des déplacements et des flux de gènes chez un mustélidé forestier, la Martre des pins (*Martes martes*). PhD thesis, Universite de Reims Champagne, Ardenne.

Barja, I. (2017). Marta – *Martes martes*. In A. Salvador & I. Barja (Eds.), *Enciclopedia Virtual de los Vertebrados Españoles*. Museo Nacional de Ciencias Naturales, Madrid.

Bissonette, J. A., Fredrickson, R.J., & Tucker, B.J. (1988). The effects of forest harvesting on marten and small mammals in western Newfoundland. Report prepared for the Newfoundland and Labrador Wildl. Div. And Corner Brook Pulp and Pup. Ltd. Utah State University.

Brainerd, S., & Rolstad, J. (1997). Habitat selection and range use by the Eurasian pine marten (*Martes martes*) in relation to commercial forestry precitces in southern boreal Scandinavia. PhD thesis, Agricultural University of Norway.

Chotolchu, N., Stubbe, M., & Dawaa, N. (1980). The stone marten *Martes foina* (Erxleben, 1777) in Mongolia. *Acta Theriologica*, *25*. doi.org/10.4098/AT.arch.80-10

Duduś, L. (2014). Biologia kuny domowej (*Martes foina* Erxleben, 1777) we Wrocławiu. PhD thesis, Institute of Nature Conservation PAS, Kraków.

Dumić, T., Bardić, L., Pintur, K., Štedul, I., & Fabijanić, N. (2016). Kraniometrijska obilježja kune bjelice (*Martes foina* ERX.) na području sjeverozapadne Hrvatske (Craniometrical characteristics of Stone marten (*Martes foina* ERX.) in northwest Croatia). 51 hrvatski i 11 međunarodni simpozij agronoma, Opatija, Hrvatska.

Fehr, M. H. (2008). Morphologie und topographische Anatomie der Brust-, Bauch- und Beckenhohlenorgane des Steinmarders (*Martes foina* Erxleben 1777). PhD thesis, Institute for Parasitology, Institute for Terrestrial and Aquatic Wildlife Research, Hannover.

Goretti, E., Pallottini, M., Goga, B. T. C., Selvaggi, R., Petroselli, C., Vercillo, F., & Cappelletti, D. (2018). Mustelids as bioindicators of the environmental contamination by heavy metals. *Ecological Indicators, 94*. doi.org/10.1016/j.ecolind.2018.07.004

Gouwy, J., Van Den Berge, K., & Berlengee en Dirk Vansevenant, F. (2013). Boommarters in Sinaai: opvolging van drie nesten. Report, Instituut voor Natuur - en Bosonderzoek, Brussel.

Herr, J. (2008). Ecology and behaviour of urban stone martens (*Martes foina*) in Luxembourg. PhD thesis, University of Sussex.

Krott, P. (1981). Erstnachweis des Steinmarders, *Martes foina* (Erxleben, 1977), im Kleinsolktal (Mammalia, Carnivora). *Mitteilungen der Abteilung fuer Zoologie am Landesmuseum Joanneum, 10.*

Krüger, H.-H. (1995). Zur Populationsstruktur und Morphologie des Baummarders (*Martes martes* L., 1758) und Steinmarders (*Martes foina* Erxl., 1777). PhD thesis, University of Göttingen.

Lachat Feller, N. (1993). Eco-éthologie de la fouine (*Martes foina* Erxleben, 1777) dans le Jura suisse. PhD thesis, University of Neuchâtel.

Liese, A., Roth, M., Engel, E., & Schley, L. (2014). Untersuchungen zur Verbreitung des Baummarders (*Martes martes*) in Luxemburg. *Bull. Soc. Nat luxemb., 115*.

Lode, T. (1991). Conspecific recognition and mating in stone marten *Martes foina*. *Acta Theriologica*, *36*. doi.org/10.4098/AT.arch.91-28

Lopez-Martin, J. M., Ruiz-Olmo, J., & Cahill, S. (1992). Autumn home range and activity of a stone marten (*Martes foina* Erxleben, 1777) in northeastern Spain. *Miscellania Zoologica (Barcelona), 16*.

López-Martín, J. (2003). Comparacion de la ecologia de la marta (*Martes martes* L. 1758) y la garduna (*M. foina* Erx. 1777) en el N.E. Iberico: interacciones con la gineta (*Genetta genetta* L. 1758). PhD thesis, Universitat de Barcelona.

Marchesi, P. (1989). Ecologie et comportament de la Martre (*Martes martes* L.) dans le Jura suisse. PhD thesis, Universite de Neuchatel.

Mergey, M. (2007). Résponses des populations de martres d'Europe (*Martes martes*) à la fragmentation de l'habitat: mécanismes comportementaux et censéquences. PhD thesis, Universite de Reims Champagne-Ardenne.

Müskens, G., & Broekhuizen, S. (2005). De steenmarter (*Martes foina*) in Borgharen: aantal, overlast en schade. Report, Centre for Ecosystem Studies, Wageningen Environmental Research (Alterra-rapport 1259).

Nicht, M. (1969). Ein Beitrag zum Vorkommen des Steinmarders, *Martes foina* (Erxleben, 1770), in der Großstadt (Magdeburg). *Zeitschrift für Jagdwissenschaft, 15*.

Nowak, A. (2010). Wykorzystanie przestrzeni przez kunę domową w centrum miasta Krakowa. PhD thesis, Jagiellonian University, Kraków.

Peeva, S. (2019). Sexual size dimorphism in stone marten (*Martes foina*, Erxl. 1777) from Sarnena Sredna gora Mts (Bulgaria). *Trakia Journal of Sciences*, *17*.

Posillico, M., Serafini, P., & Lovari, S. (1995). Activity patterns of the stone marten *Martes foina* Erxleben, 1777, in relation to some environmental factors. *Hystrix*, *7*.

Roedel, H. G., & Stubbe, M. (2006). Shifts in food availability and associated shifts in space use and diet in stone marten. *Lutra, 49*.

Schinzel, B. (1998). Radiotelemetrie- und GIS-Analysen zum Raum-Zeit-Verhalten von Baum- (*Martes martes*) und Steinmardern (*Martes foina*) im Untersuchungsgebiet Wahlen. PhD thesis, Universität des Saarlandes.

Schröpfer, R., Biedermann, W., & Szczesniak, H. (1989). Seasonal changes of the home range in the pine marten *Martes martes* (in German). In M. Stubbe (Ed.), *Populationsokologie marderartiger Säugetiere*. Martin-Luther Universität, Halle.

Stier, N. (2012). Zur Populationsökologie des Baummarders (*Martes martes* L., 1758) in Nordost-Deutschland. Wildtierforschung in Mecklenburg-Vorpommern.

Stier, N., Borchert, M., Meißner-Hylanová, V., Pinnecke, J., Schmüser, H., Hoffmann, D., Eckern, S., Häger, B., & Roth, M. h. (2015). Erfassungsmethoden von Baummarder und Iltis zur Beurteilung ihrer Populationszustände. Report.

Storch, I. (1988). Zur Raumnutzung von Baummardern. *Zeitschrift für Jagdwissenschaft, 34*.

Figure A1. Pine marten (*Martes martes*) body masses data distribution over time in European countries (only data used to statistical modelling). The number of sample size in parentheses.

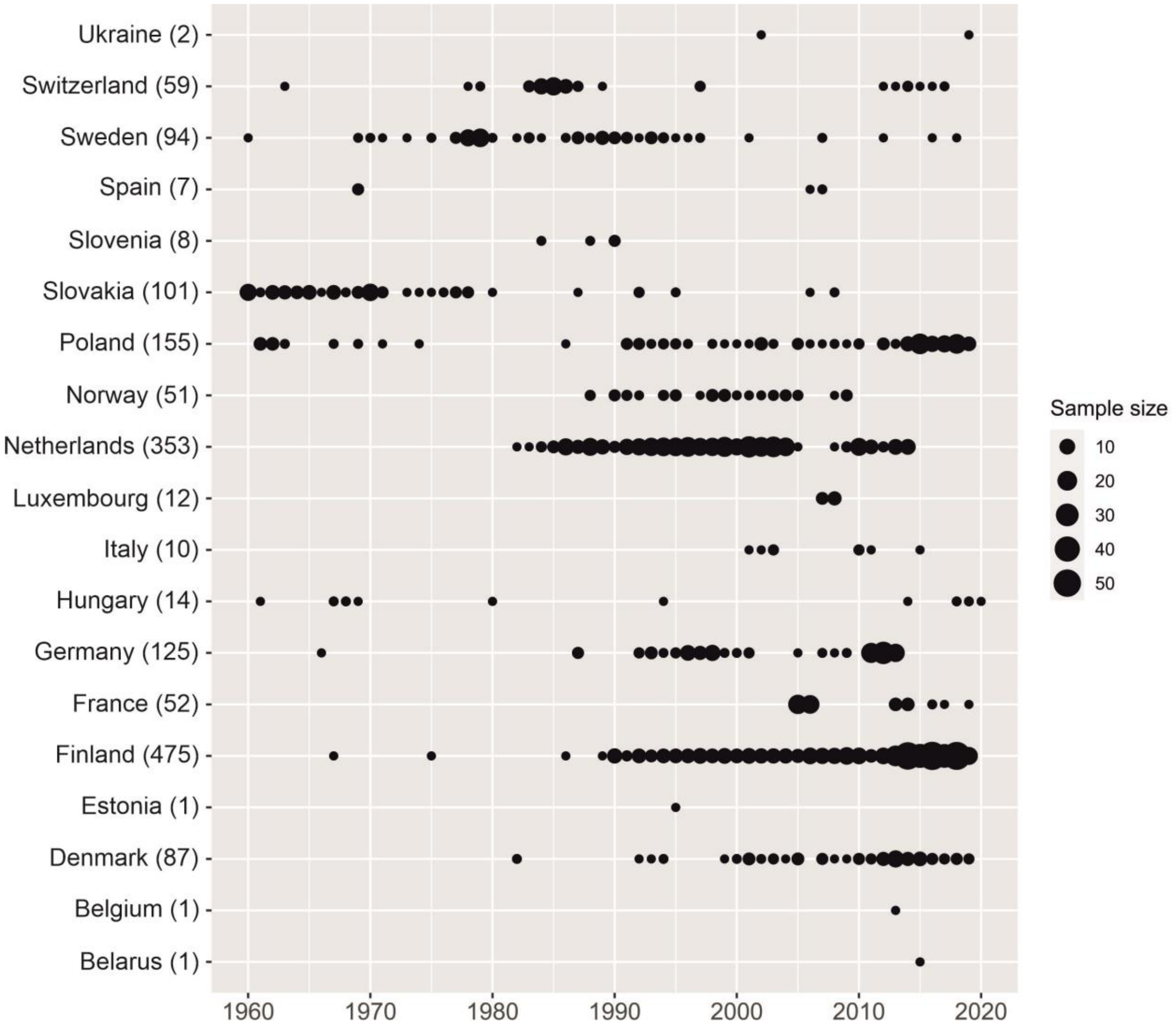

Figure A2. Spatial distribution of pine marten (*Martes martes*) data used to modelling along its European range.

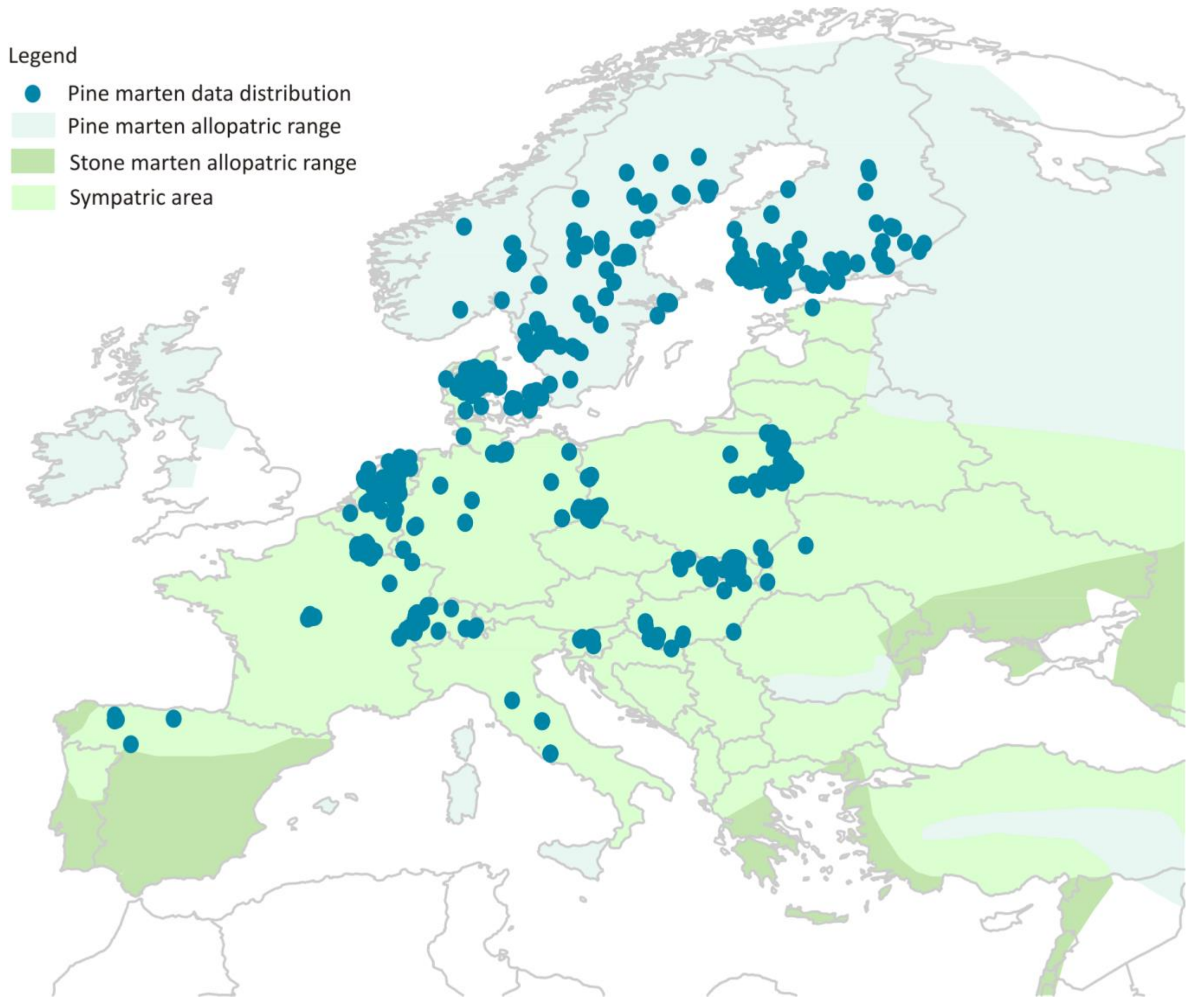

Figure A3. Stone marten (*Martes foina*) body masses data distribution over time in European countries (only data used to statistical modelling). The number of sample size in parentheses.

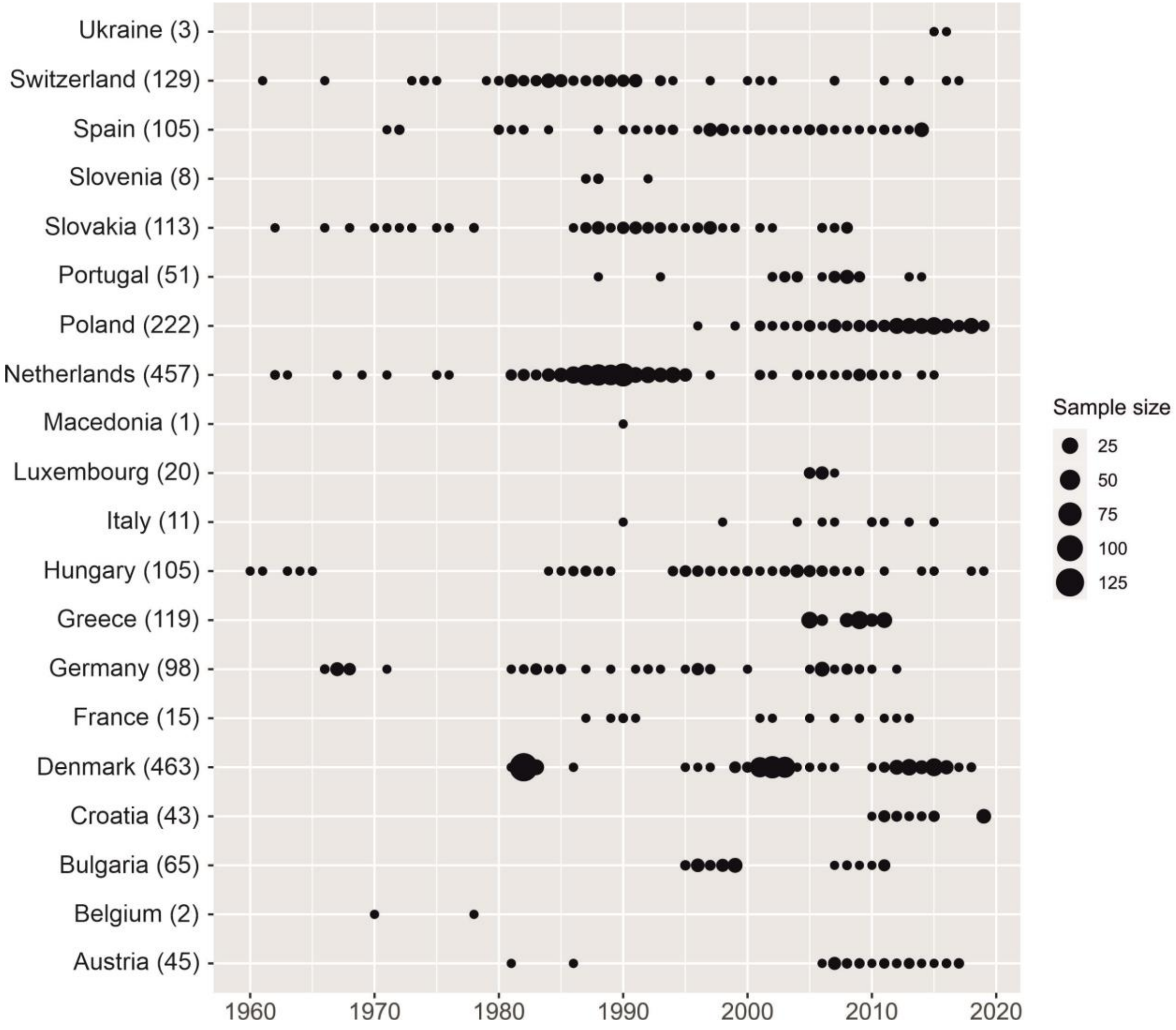

Figure A4. Spatial distribution of stone marten (*Martes foina*) data used to modelling along its European range.

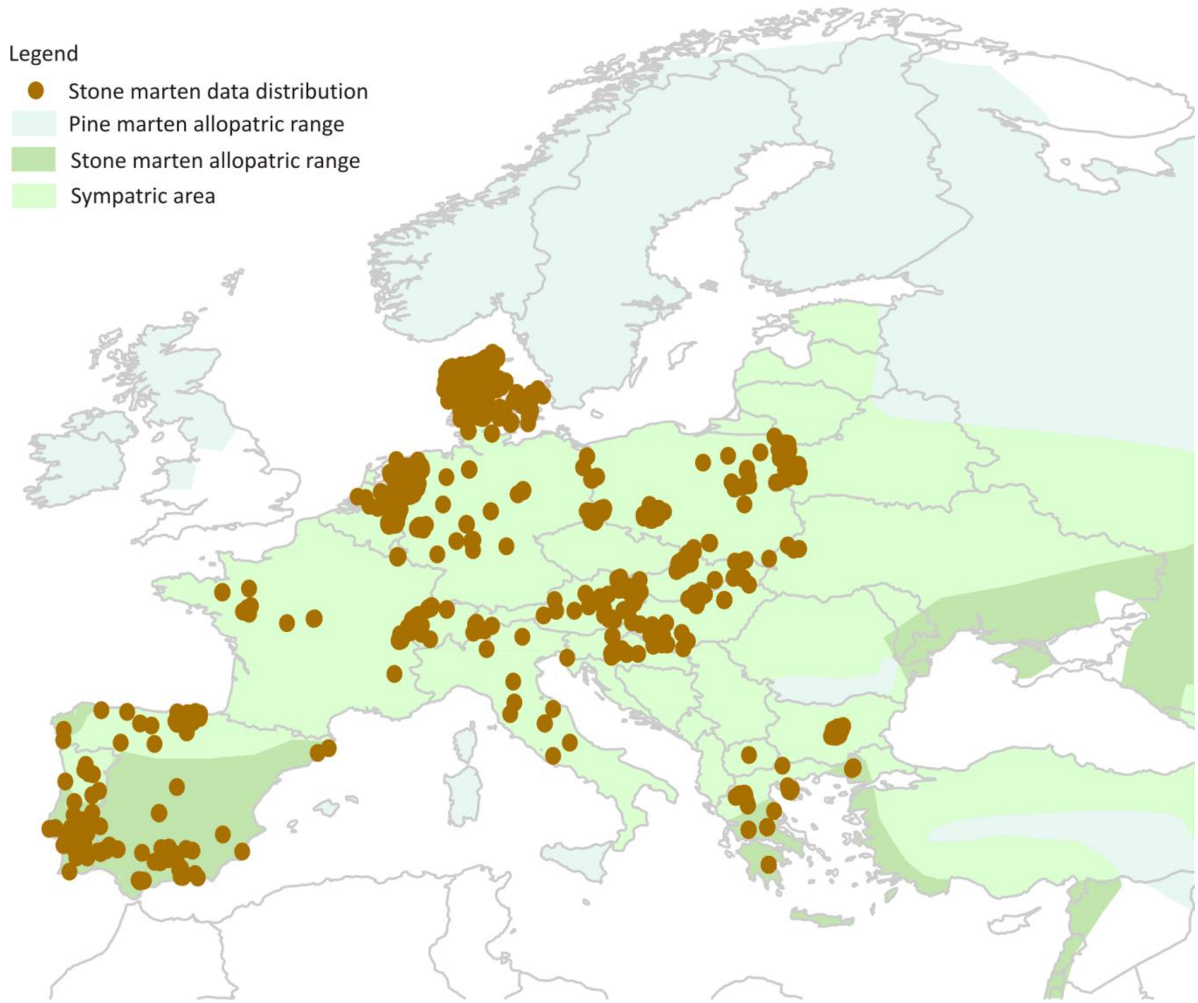

Figure A5. Month and sex components for pine and stone marten. The graphics show the estimated of GAMs smoother effects with 95% confidence intervals in grey, where negative trends yield decrease body weight with increasing time in months.

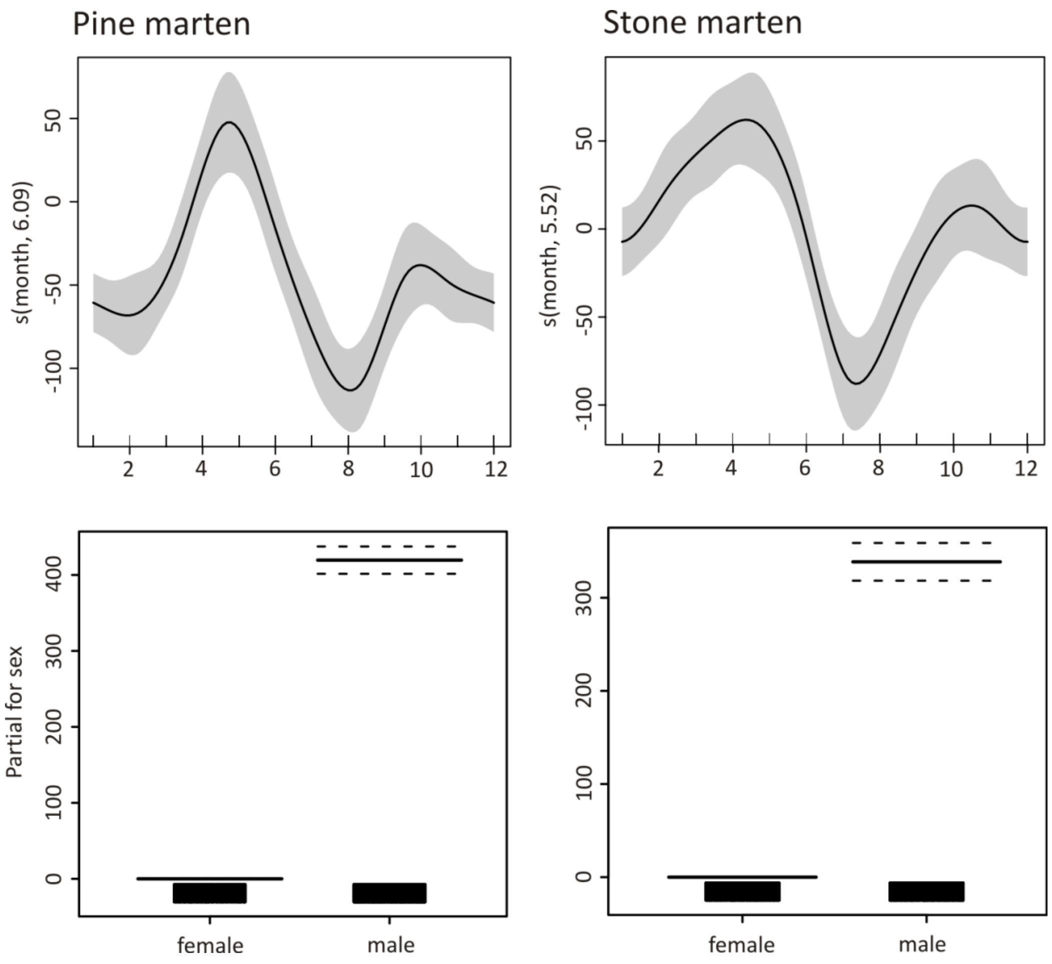

Table A2. Body mass of pine and stone marten predicted by GAMs and average increase of body mass over 59 years in sympatric and allopatric sites of the geographical net with resolution 5° x 5° of latitude and longitude (sites location in Fig. 3). Sites S15 and S16 were analyzed since 1991 due to late stone marten expansion in north-eastern Europe, while S5 and S6 were analyzed since 1980 due to a lack of data before that time.

Site	N	E	Sympatric/ allopatric area	Predicted body mass averaged for years (g)		Average increase of body weight over time (%)
				Males	Females	
Pine marten						
S2	42.5	-5	sympatric	1681.6	1262.1	18.7
S4	42.5	10	sympatric	1475.0	1013.6	62.3
S7	47.5	0	sympatric	1433.1	1135.7	13.2
S8	47.5	5	sympatric	1526.3	1106.8	31.1
S9	47.5	10	sympatric	1484.6	1055.5	42.1
S10	52.5	5	sympatric	1531.1	1111.6	18.5
S11	52.5	10	sympatric	1459.5	1062.1	27.6
S12	47.5	15	sympatric	1433.9	1014.4	32.8
S13	47.5	20	sympatric	1394.6	973.6	19.3
S14	52.5	15	sympatric	1430.6	1017.0	21.6
S15	52.5	20	sympatric	1681.6	975.1	15.5
S16	52.5	25	sympatric	1262.7	878.0	27.1
S17	57.5	25	allopatric	1384.9	843.2	22.4
S18	57.5	15	allopatric	1297.5	1011.4	29.2
S19	57.5	10	sympatric	1375.5	1040.0	23.6
S20	62.5	10	allopatric	1393.1	956.0	5.3
S21	62.5	15	allopatric	1279.1	965.4	17.6
S22	62.5	25	allopatric	1555.2	859.6	11.1
Stone marten						
S1	37.5	-5	allopatric	1578.7	1240.1	-10.1
S2	42.5	-5	sympatric	1490.8	1152.2	2.6
S3	42.5	0	sympatric	1541.7	1203.1	-8.0
S4	42.5	10	sympatric	1567.5	1228.9	10.9
S5	42.5	20	sympatric	1607.7	1269.1	64.1
S6	42.5	25	sympatric	1614.7	1276.1	106.9
S7	47.5	0	sympatric	1579.9	1241.3	14.7
S8	47.5	5	sympatric	1633.8	1295.2	11.3
S9	47.5	10	sympatric	1598.7	1260.1	9.8
S10	52.5	5	sympatric	1606.9	1268.3	23.9
S11	52.5	10	sympatric	1574.8	1236.2	1.4
S12	47.5	15	sympatric	1556.1	1217.5	12.0
S13	47.5	20	sympatric	1550.9	1212.3	17.2
S14	52.5	15	sympatric	1542.8	1204.2	-13.1
S15	52.5	20	sympatric	1561.4	1222.8	3.6

S16	52.5	25	sympatric	1604.2	1265.6	-5.2
S19	57.5	10	sympatric	1610.8	1272.2	8.7

Table A3. Body weight ratio PM/SM averaged for years and sexes, predicted by GAMs. Sites S15 and S16 were analyzed since 1991 due to late stone marten expansion in north-eastern Europe.

	S2	S4	S7	S8	S9	S10	S11	S12	S13	S14	S15	S16	S19
average	1.13	0.92	0.98	0.93	0.92	0.96	0.94	0.92	0.90	0.93	0.93	0.88	0.91
min	1.04	0.75	0.94	0.87	0.80	0.90	0.81	0.82	0.87	0.73	0.91	0.85	0.83
max	1.19	1.08	1.03	1.03	0.99	1.02	0.99	0.97	0.92	1.01	0.97	0.96	0.95
